# Responses of Withdrawal Interneurons to Serotonin Applications in Naïve and Learned Snails Are Different

**DOI:** 10.3389/fncel.2017.00403

**Published:** 2017-12-14

**Authors:** Tatiana K. Bogodvid, Vyatcheslav V. Andrianov, Irina B. Deryabina, Lyudmila N. Muranova, Dinara I. Silantyeva, Aliya Vinarskaya, Pavel M. Balaban, Khalil L. Gainutdinov

**Affiliations:** ^1^Laboratory of Neuroreabilitation of Motor Disorders, Institute of Fundamental Medicine and Biology, Kazan Federal University, Kazan, Russia; ^2^Department of Biomedical Sciences, Volga Region State Academy of Physical Culture, Sport and Tourism, Kazan, Russia; ^3^Laboratory of Cellular Neurobiology of Learning, Institute of Higher Nervous Activity and Neurophysiology, Russian Academy of Sciences, Moscow, Russia

**Keywords:** serotonin, 5-hydroxytryptophan, associative learning, identified neurons, membrane potential, threshold potential, snail

## Abstract

Long-term changes in membrane potential after associative training were described previously in identified premotor interneurons for withdrawal of the terrestrial snail *Helix*. Serotonin was shown to be a major transmitter involved in triggering the long-term changes in mollusks. In the present study we compared the changes in electrophysiological characteristics of identifiable premotor interneurons for withdrawal in response to bath applications of serotonin (5-HT) or serotonin precursor 5-hydroxytryptophan (5-HTP) in preparations from naïve, neurotoxin-injected or associatively trained snails. It was found that 5-HT or 5-HTP applications caused a significant decrease of membrane potential in premotor interneurons of naïve snails, associatively trained snails and snails with impaired serotonergic system by injection of a selective neurotoxin 5,7-dihydroxytryptamine (5,7-DHT) 1 week before the experiments. Applications of 5-HT or 5-HTP did not cause significant changes in the action potential (AP) threshold potential of these neurons in naïve snails. Conversely, applications of 5-HT or 5-HTP to the premotor interneurons of previously trained or 5,7-DHT-injected snails caused a significant increase in the firing threshold potential in spite of a depolarizing shift of the resting membrane potential. Results demonstrate that responsiveness of premotor interneurons to extracellularly applied 5-HT or 5-HTP changes for days after the associative training or serotonin depletion. Similarity of the effects in trained and 5,7-DHT-injected animals may be due to massive release of serotonin elicited by 5,7-DHT injection. Our results suggest that serotonin release due to aversive conditionining or elicited by the neurotoxin administration triggers similar changes in resting membrane potential and AP threshold in response to bath applications of 5-HT or its precursor 5-HTP.

## Introduction

Questions concerning the mechanisms of learning and memory are still open. Experimental data show that the cellular processes associated with learning occur at least at two levels: long-term modifications of the synaptic transmission efficiency (presynaptic level) and changes in the endogenous properties of the whole neuron and its membrane potential (postsynaptic level; Balaban, 2002, 2017; Crow, 2004; Hawkins et al., 2006; Jin et al., 2009; Saar and Barkai, 2009; Gainutdinov et al., 2011; Hu et al., 2011; Vasil’yeva et al., 2015). Several specific tasks can be distinguished within the problem of cellular mechanisms of learning. These tasks include an analysis of the excitability of both presynaptic and postsynaptic structures, i.e., membrane characteristics of neurons and synaptic transmission (Alkon, 1984; Byrne, 1987; Frysztak and Crow, 1997; Bao et al., 1998; Cleary et al., 1998; Gainutdinov et al., 1998; Daoudal and Debanne, 2003; Gainutdinova et al., 2003; Mozzachiodi et al., 2008; Crow and Tian, 2009; Debanne and Poo, 2010; Glanzman, 2010; Mozzachiodi and Byrne, 2010; Gainutdinov et al., 2011; Cavallo et al., 2014a,b; Pivovarov et al., 2014), and in particular the role of persistent sodium current in mechanisms of neuronal plasticity (Kiss, 2003; Nikitin et al., 2006; Kiss et al., 2009; Nagakura et al., 2010). One of the most interesting aspects is the study of neuromodulatory processes (Sakharov, 1990, 2012; Whitaker-Azmitia, 1999; Gillette, 2006; Hernádi et al., 2008; Ierusalimskii and Balaban, 2010; Hart et al., 2011; Nikitin et al., 2013; Balaban et al., 2014), which are necessary for the formation of long-term memory. Serotonin (5-HT) is a major neuromodulator for the nervous system of mollusks. It has been found that 5-HT is the main transmitter that modulates the withdrawal behavior in mollusks and it is necessary for the associative memory formation (Kandel and Schwartz, 1982; Balaban et al., 1987; Gainutdinov et al., 1999; Pavlova, 2001; Burrell and Sahley, 2005; D’iakonova, 2007; Il-Han et al., 2010; Hirayama et al., 2014; Dyakonova et al., 2015). Recently (Ierusalimskii and Balaban, 2010; Sakharov, 2012) the concept concerning that the volume (extra-synaptic) transmission of 5-HT plays a major role in the mechanisms of memory in mollusks was discussed. The activity of identified modulatory serotonergic neurons of the pedal ganglion was shown to serve as a reinforcement for associative facilitation of glutamatergic synaptic inputs in the terrestrial snail (Balaban et al., 2001). In snails with impaired 5-HT neurons both aversive learning and reconsolidation were impaired, suggesting a necessity of 5-HT for behavioral plasticity (Balaban et al., 2016).

In addition to the well-known role of 5-HT as a transmitter, it has been shown that after secretion into the extracellular environment it can realize integrative functions (Sakharov, 1990; Zakharov et al., 1995; Levenson et al., 1999; Liao et al., 1999; Marinesco et al., 2004; Timoshenko et al., 2014). These results supported usage of the 5-HT bath applications as a reinforcing stimulus in the models of cellular analogs of learning. Application of 5-HT causes effects similar to the effects of sensitizing stimuli in the neural network underlying the withdrawal responses, which suggests that electrophysiological correlates of plasticity can be reproduced by bath 5-HT applications (Farley and Wu, 1989; Clark and Kandel, 1993; Sugita et al., 1997; Malyshev et al., 1998; Mauelshagen et al., 1998; Shevelkin et al., 1998; Sun and Schacher, 1998; Bailey et al., 2000; Fickbohm et al., 2005; Fioravante et al., 2007; Hernádi et al., 2008; Lin et al., 2010; Hart et al., 2011; Hu et al., 2011). It has been shown that applications of 5-HT lead to broadening of action potentials (APs) in sensory neurons mediated by the 5-HT_1_ receptors that are blocked by the antagonist of 5-HT receptor methiothepin (Dumitriu et al., 2006). It was also shown that antagonist of 5-HT receptors mianserin blocked two forms of withdrawal behavior of *Lymnaea* caused by an unconditioned stimulus (extract of crayfish tissue), and another 5-HT receptor antagonist methylsergide blocked formation of the long-term memory after training (Il-Han et al., 2010). The 5-HT_1_ receptors were described for the soma of identified premotor interneurons of the terrestrial snail, which are the object of our study (Pivovarov and Nistratova, 2003; Abramova et al., 2006).

In the present study we carried out a comparative investigation of changes of the excitability of the premotor interneurons in response to the applications of 5-HT or the serotonin precursor 5-HTP in preparations from naive or trained snails. In addition, we carried out studies of the changes of the excitability of the premotor interneurons in response to applications of 5-HT or its precursor 5-hydroxytryptophan (5-HTP) in preparations from snails with an impaired 5-HT system by injection of the selective neurotoxin 5,7-dihydroxytryptamine (5,7-DHT). Previously it was shown that snails with impaired serotonergic system cannot acquire aversive memory (Balaban et al., 1987; Glanzman et al., 1989; Gainutdinov et al., 1999; Andrianov et al., 2015).

## Materials and Methods

### Experimental Animals

Terrestrial snails *Helix lucorum* of the Crimean population were used for experiments. The snails were stored in a dormant state in a fridge. Prior to the experiments, the snails were kept active for at least 2 weeks in glass terrariums in a humid atmosphere at room temperature, with excess of food). All experimental procedures were conducted in accordance with the guidelines for the care and use of laboratory animals published by the National Institutes of Health and Directive 2010/63/EC of the European Parliament and Council of Europe on September 22, 2010. All groups were housed in separate terrariums that were stored together in the same room under the same conditions. Electrophysiological measurements were performed on isolated nervous system preparations the next day after the end of training. Prior the dissection procedure, the snails were anesthetized by immersion for 30 min in ice-water mixture.

### Withdrawal Reflex Conditioning

A withdrawal conditioned reflex to the tap on the shell was induced in half of the snails (detailed procedure see Gainutdinova et al., 2003; Andrianov et al., 2015). Briefly, a conditioned stimulus (CS) was a light tapping on the shell (2 times) with a pencil, which normally did not cause a withdrawal response in the animal. A puff of the air into the opening of the pulmonary cavity (pneumostome) was used as an unconditioned stimulus, which caused an unconditioned withdrawal response—closure of the pneumostome—that was video recorded. Combinations of stimuli (delay between tapping and air puff was 1 s) were presented at intervals of 2–4 min. The associative conditioned response was induced in 3 days as a result of presentation of about 150 pairings of conditioned and unconditioned stimuli. The result of this training was a complete closure of the pneumostome in response to a CS, which was scored as a positive reaction. The reflex was considered fully conditioned when the closure of the pneumostome in response to the CS occurred in 100% in a session of 10 presentations of CS.

### Intracellular Recording

Analysis of electrical characteristics was carried out in the readily identifiable giant premotor interneurons (#3) of the withdrawal reflex located in the rostral part of parietal ganglia (description and map in Balaban, 2002). The isolated nervous system was placed in a saline solution (SS) of the following composition: NaCl-80 mM, KCl-4 mM, CaCl_2_-10 mM, MgCl_2_-6 mM, NaHCO_3_-5 mM (or Tris-5 mM); pH-7.6–7.8. Measurements were carried out at room temperature (18–21°C) using intracellular glass microelectrodes filled with 2.5 M KCl and having a resistance of 10–30 MΩ. Since the premotor giant interneurons LPa3 and RPa3 are normally silent, a single-electrode method of cell stimulation was used. To trigger the AP in an isolated preparation through a recording microelectrode, a current pulse lasting 1 s was applied to the cell using a bridge curcuit for compensation of the constant current. A minimum current required to generate 3–5 APs was used. The following parameters of nerve cells were recorded: membrane potential (Vm; the value immediately before the onset of the stimulus pulse) and AP threshold (Vt). The threshold potential was scored as the difference between the membrane resting potential and the potential value during AP’s generation at which its rate of increase (the first derivative of the potential with respect to time) reached a certain value of 1 V/s (shown by Khodorov, 1974 and described in details in Andrianov et al., 2015).

### Drugs

In the main series of experiments, we studied responses of the premotor interneurons Lpa3 and RPa3 to exposure of the nervous system of naïve or trained snails for 30 min via bath application of the following substances:

1. Serotonin (5-HT) at a final bath concentration of 4 × 10^−5^ M.
2. 5-hydroxytryptophan (5-HTP), a precursor of the synthesis of serotonin at a final bath concentration of 4 × 10^−5^ M.
3. In a separate series of experiments, we have studied the responses of the premotor interneurons LPa3 and RPa3 to applications of 5-HT or 5-HTP (bath application for 30 min) in preparations from the snails in which the serotonergic neurons were selectively impaired by injection of a neurotoxic analog of serotonin, 5,7-dihydroxytryptamine (5,7-DHT, details in Balaban et al., 1987). Briefly, 5,7-DHT dissolved in 0.1 ml of SS with ascorbic acid in concentration of 0.1% as an antioxidant was administered once in a dose of 20 mg/kg of body weight. Testing of responses to 5-HT or 5-HTP applications to the isolated nervous system of the snail was performed a week after the injection of 5,7-DHT.

### Data Analyses

The results are shown as mean ± SEM. One-way ANOVA followed by Tukey *post hoc* test, paired and unpaired *t*-test, and non-parametric Mann–Whitney test were used for comparison between two groups. Statistical software SigmaStat32 was used. The statistical significance criterion was *p* < 0.05.

## Results

### Changes in the Membrane Potential and Threshold of AP Generation in Premotor Interneurons of Withdrawal Behavior LPa3 and RPa3 in Naïve Snails in Response to Bath Applications of Serotonin or the Precursor 5-Hydroxytryptophan

**Figure 1:**
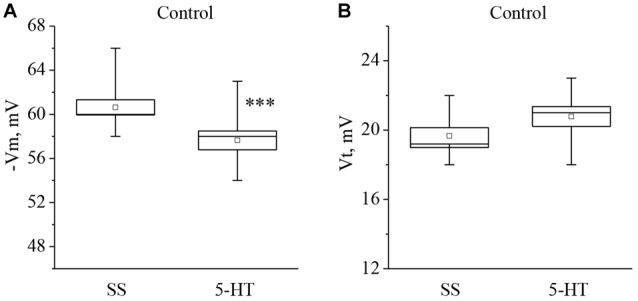
Effect of bath application of serotonin (5-HT) in concentration 4 × 10^−5^ M on the membrane potential (**A**, −Vm, *n* = 11) and the threshold potential (**B**, Vt, *n* = 9) of the premotor interneurons of withdrawal withdrawal behavior LPa3 and RPa3 in naive snails. Mean (open rectangle) and SEM (box), as well as median (line in a box) values are shown for each data group, whiskers show range of data (min-max). 5-HT—application of serotonin, SS—saline solution. Vertical axis shows values of potential, in mV. Asterisks (***) indicate significant difference (*p* < 0.001, independent *t*-test).

Recording of electrical characteristics of the neurons from naïve snails showed that the mean resting membrane potential (Vm) of the giant premotor interneurons of withdrawal behavior was −60.6 ± 0.7 mV (*n* = 11), and the mean threshold potential (Vt) was 19.7 ± 0.5 mV (*n* = 9). A significant long-term decrease of Vm to −57.6 ± 0.8 mV, (*n* = 11) and a non-significant increase of Vt to 20.8 ± 0.6 mV (*n* = 9) was observed after bath application of 5-HT to the isolated nervous system of naive snails (Figure 1).

**Figure 2:**
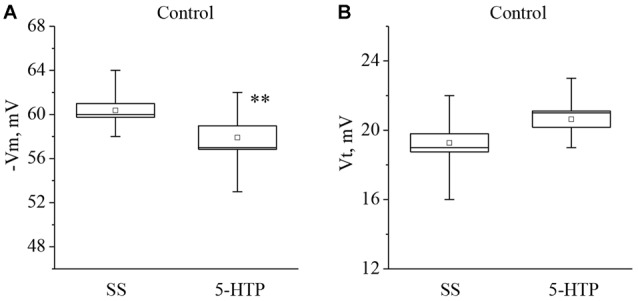
The effect of bath application of the precursor of the serotonin synthesis 5-HTP in concentration 4 × 10^−5^ M on the membrane potential (**A**, −Vm, *n* = 11) and the threshold potential (**B**, Vt, *n* = 10) of the premotor interneurons of withdrawal behavior withdrawal LPa3 and RPa3 in naive snails. Mean (open rectangle) and SEM (box), as well as median (line in a box) values are shown for each data group, whiskers show range of data (min-max). 5-HTP—application of precursor of the serotonin synthesis, SS—saline solution. Vertical axis shows values of potential, in mV. Asterisks (**) indicate significant difference (*p* < 0.01, independent *t*-test and One-way ANOVA).

Similar changes were observed after application of 5-HTP (Figure 2). The mean membrane potential decreased from −60.3 ± 0.6 mV to −57.9 ± 1.1 mV (*n* = 11). The mean threshold potential generation nonsignificantly increased from 19.4 ± 0.5 mV to 20.6 ± 0.5 mV (*n* = 10).

### Effects of Associative Training on the Membrane Potential and Threshold of AP Generation in Premotor Interneurons of Withdrawal Behavior LPa3 and RPa3

**Figure 3:**
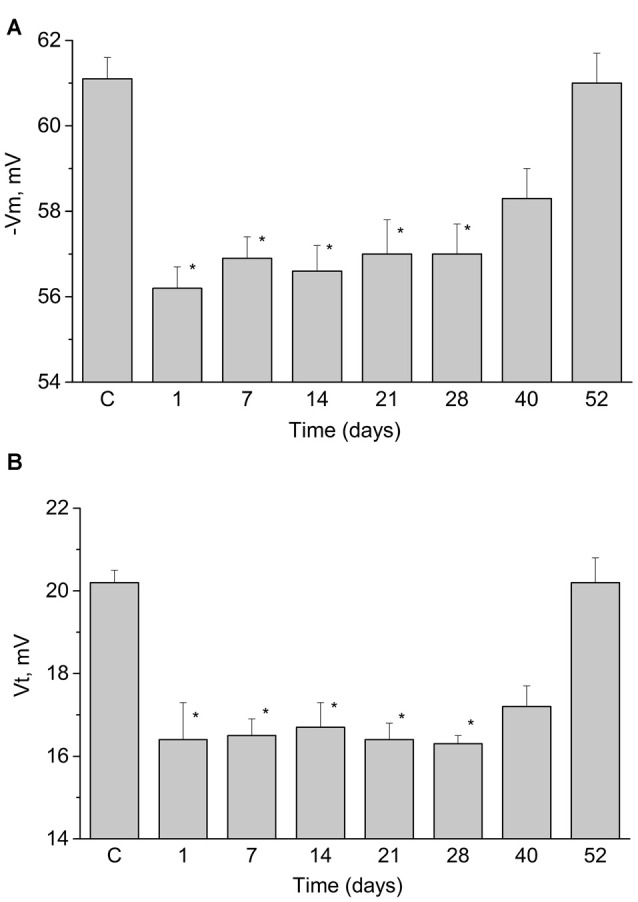
Duration of changes in resting membrane potential (**A**, −Vm) and the threshold potential (**B**, Vt) of the premotor interneurons of withdrawal behavior LPa3 and RPa3 at different times after the end of training session (marked under the bars). Each bar represents mean ± SEM values for different groups of snails (*n* = 6 or more in each). Asterisk (*) indicate significant difference (*p* < 0.05, One-way ANOVA with *post hoc* paiwise comparison with the control values (C)).

It was described earlier (Gainutdinov et al., 1998, 2000) that associative conditioning of the terrestrial snail *Helix* elicited long-lasting changes in the electrical characteristics of functionally identified premotor interneurons triggering withdrawal behavior. We have measured the duration of retention of long-lasting changes (Figures 3A,B) in these neurons in eight groups of trained snails at different time points. We have observed significant changes in the resting membrane potential and threshold of AP generation that lasted for 40 days after the end of training (Figures 3A,B). These results demonstrate that the excitability of LPa3 and RPa3 in trained animals increased relative to that in preparations from the naive animals.

### Changes in the Membrane Potential and AP Threshold in Premotor Interneurons LPa3 and RPa3 from Trained Snails in Response to Bath Applications of Serotonin or 5-Hydroxytryptophan

The mean resting membrane potential of the identified premotor interneurons in the trained snails appeared to be lower than in naïve animals by approximately 4 mV, and the threshold of APs generation also was lower by approximately 2 mV (Figures 3A,B). In the next series of experiments we tested whether the responses to 5-HT bath applications differed in neurons from the trained animals.

**Figure 4:**
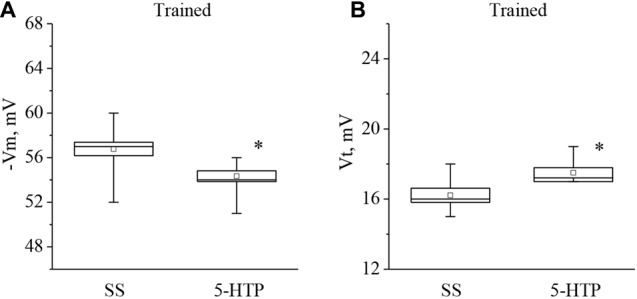
Effect of bath application of serotonin (5-HT) in concentration 4 × 10^−5^ M on the membrane potential (**A**, −Vm, *n* = 13) and the threshold potential (**B**, Vt, *n* = 9) of premotor interneurons of withdrawal behavior withdrawal LPa3 and RPa3 in trained snails. Mean (open rectangle) and SEM (box), as well as median (line in a box) values are shown for each data group, whiskers show range of data (min-max). 5-HT—application of serotonin, SS—saline solution (before application of serotonin). Vertical axis shows values of potential, in mV. Asterisks (*) indicate significant difference (*p* < 0.05, independent *t*-test and One-way ANOVA).

It was found that both 5-HT application, or 5-HTP bath application to the isolated nervous system of the trained snails caused a significant additional decrease in the membrane potential and, surprisingly, a significant increase in the threshold potential. The mean membrane potential decreased from −56.2 ± 0.3 mV to −52.1 ± 0.8 mV (*n* = 13) and the mean threshold potential increased from 16.4 ± 0.7 mV to 19.0 ± 0.9 mV (*n* = 6) after application of 5-HT (Figure 4).

**Figure 5:**
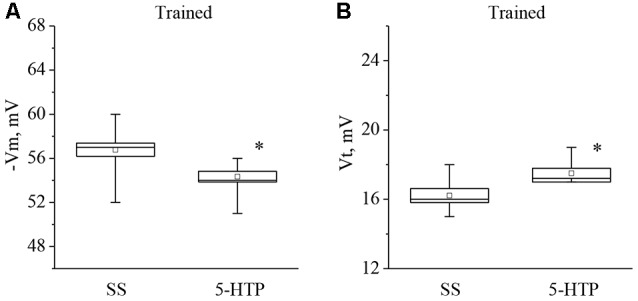
Effect of bath application of the precursor of the serotonin synthesis (5-HTP) in concentration 4 × 10^−5^ M on the membrane potential (**A**, −Vm, *n* = 9) and the threshold potential (**B**, Vt, *n* = 8) of premotor interneurons of withdrawal behavior withdrawal LPa3 and RPa3 in trained snails. Mean (open rectangle) and SEM (box), as well as median (line in a box) values are shown for each data group, whiskers show range of data (min-max). 5-HTP—application of precursor of the serotonin synthesis, SS—saline solution (before application of precursor of the serotonin synthesis). Vertical axis shows values of potential, in mV. Asterisk (*) indicate significant difference (*p* < 0.05, independent *t*-test and Mann–Whitney test).

Moreover, the mean membrane potential decreased from −56.7 ± 0.8 mV to −54.3 ± 0.6 mV (*n* = 9) and the mean threshold potential increased from 16.2 ± 0.4 mV to 17.5 ± 0.3 mV (*n* = 8) after application of 5-HTP (Figure 5). These results demonstrate that the responses to 5-HT/5-HTP bath applications in trained animals are more pronounced relative to preparations from the control animals.

### Changes in the Membrane Potential and AP Threshold in Response to Bath Applications of Serotonin or 5-Hydroxytryptophan in Premotor Interneurons LPa3 and RPa3 in Preparations from Snails Injected with 5,7-DHT

In the next series of experiments we determined whether the responses to 5-HT and 5- HTP changed due to 5-HT depletion. Preparations were made from snails given an injection of the selective neurotoxin 5,7-DHT, which is known to deplete significantly the production of 5-HT in serotonergic neurons without affecting the serotonin receptors (for review see Balaban, 2002).

**Figure 6:**
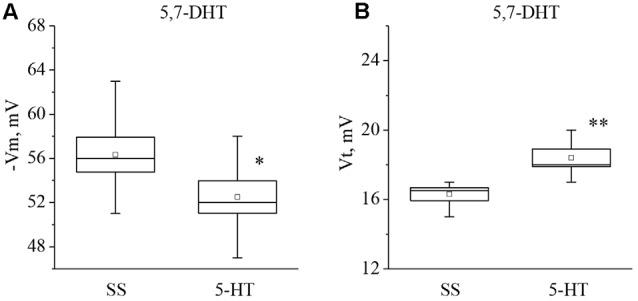
Effect of application of serotonin (5-HT) in concentration 4 × 10^−5^ M into washing solution on the membrane potential (**A**, −Vm, *n* = 6) and the threshold potential (**B**, Vt, *n* = 6) of premotor interneurons of withdrawal behavior withdrawal LPa3 and RPa3 in snails, injected by 5,7-DHT. Mean (open rectangle) and SEM (box), as well as median (line in a box) values are shown for each data group, whiskers show range of data (min-max). 5-HT—application of serotonin, SS—saline solution (before application of serotonin). Vertical axis shows values of potential, in mV. Asterisks (*,**) indicate significant difference (*p* < 0.05, *p* < 0.01, independent *t*-test and One-way ANOVA).

**Figure 7:**
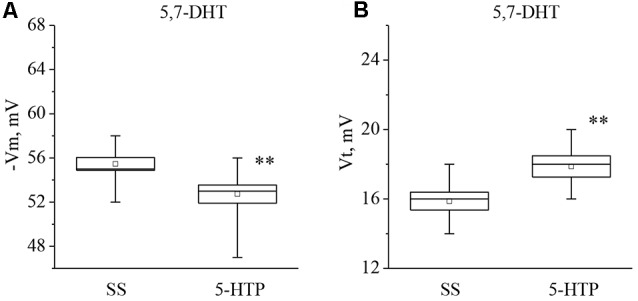
Effect of bath application of the precursor of serotonin synthesis (5-HTP) in concentration 4 × 10^−5^ M on the membrane potential (**A**, −Vm, *n* = 11) and the threshold potential (**B**, Vt, *n* = 11) of premotor neurons of withdrawal behavior withdrawal LPa3 and RPa3 in snails, injected by 5,7-DHT. Mean (open rectangle) and SEM (box), as well as median (line in a box) values are shown for each data group, whiskers show range of data (min-max). 5-HTP—application of precursor of the serotonin synthesis, SS—saline solution (before application of precursor of the serotonin synthesis). Vertical axis shows values of potential, in mV. Asterisks (**) indicate significant difference (*p* < 0.01, independent *t*-test and One-way ANOVA).

It was found that neurons from the 5,7-DHT-injected snails exhibit a resting potential and threshold potential very similar to the ones in trained snails (Figures 6, 7). This finding may be a result of massive release of serotonin known to happen due to injection of 5,7-DHT (Balaban et al., 1987).

It was observed that the membrane potential of the neurons LPa3 and RPa3 from snails injected by 5,7-DHT decreased from −56.3 ± 1.6 mV to −52.5 ± 1.5 mV (*n* = 6), and the threshold potential increased from 16.3 ± 0.4 mV to 18.4 ± 0.5 mV (*n* = 6) in response to application of 5-HT (Figure 6). Similar changes were observed after applications of 5-HTP. The membrane potential decreased from −55.5 ± 0.6 mV to −52.8 ± 0.8 mV (*n* = 11) and the threshold potential increased from 15.9 ± 0.5 mV to 17.9 ± 0.6 mV (Figure 7). Thus, in spite of the impaired function of serotonergic neurons, the changes in response to bath applications of 5-HT or 5-HTP were even higher in neurons from 5,7-DHT-injected snails.

## Discussion

Serotonin syndrome, a potentially fatal condition associated with increased serotonergic activity in the central nervous system, has attracted significant attention in recent years. (Greenier et al., 2014). Serotonin syndrome is often characterized by a triad of symptoms, which include altered mental status, neuromuscular hyperactivity and autonomic instability or hyperactivity (Boyer and Shannon, 2005; Greenier et al., 2014). The increased concentration of extracellular serotonin can be caused by blockade of the membrane transporter mechanism (Riad et al., 2004; Haberzettl et al., 2013; Leon-Pinzon et al., 2014). This condition often occurs due to the treatment of anxiety and major depression with antidepressants (Albert et al., 2014); it can also be caused by a combination of two or more serotonin-enhancing drugs (Haberzettl et al., 2013).

On the other hand, it is known that 5-HT is one of the main neuromodulatory transmitters (Sakharov, 1990; Pavlova, 2001; Gillette, 2006; D’iakonova, 2007; Hernádi et al., 2008; Ierusalimskii and Balaban, 2010; Hirayama et al., 2014). In addition to this well-known role, 5-HT has been shown to underlie integrative functions and influence the behavior when it is released from varicosities into the extracellular environment (Liao et al., 1999; Balaban et al., 2001; Jing and Gillette, 2003; Marinesco et al., 2004; Hernádi et al., 2008; Shevelkin et al., 2009; Grinkevich and Vorobiova, 2014). Recently, the importance of extrasynaptic serotonergic transmission in memory has been recognized (Ierusalimskii and Balaban, 2010; Sakharov, 2012; Hirayama et al., 2014). Research by D. A. Sakharov showed that dynamic fluctuations of levels of monoamines (primarily 5-HT in mollusks) in the local extracellular environment can determine the physiological properties and receptor profiles of individual neurons and the character of their self-organization into a pattern-generating ensemble (Sakharov, 1990, 2012; Dyakonova et al., 2015).

The presence of different types of 5-HT receptors in the nervous system of the terrestrial snail was demonstrated in a study by Kiss et al. (2003). Furthermore, 5-HT_1_-like serotonin receptors were found on the soma of premotor interneurons of the terrestrial snail used in our study (Pivovarov and Nistratova, 2003; Abramova et al., 2006; Pivovarov et al., 2014). It was also shown that various subtypes of 5-HT receptors are present in the nervous system of *Aplysia* (Barbas et al., 2003). Finally, a 5-HT receptor (apAC1), which stimulates the cAMP production and whose inhibition blocks synaptic facilitation of the sensorimotor synapse of *Aplysia*, was described (Lee et al., 2009).

Prolonged synaptic activity can cause a significant amount of 5-HT to be released into the extra-synaptic environment (Leon-Pinzon et al., 2014). Serotonergic neurons innervate brain areas where specific generators of patterns are located in mollusks and leeches (Gillette, 2006). In vertebrates it was shown that 5-HT regulates the spike activity of the principal cells of the subiculum by inhibiting a Ca^2+^ current (Petersen et al., 2017). When examining the cellular correlates of the effects of the selective serotonin reuptake inhibitor fluoxetine in rats, it was shown that an increase in anxiety is accompanied by an increase in the excitability of certain neurons (Ravinder et al., 2011).

The serotonergic system has also been found to play an important role in the modulation of stress-induced excitability and in the withdrawal behavior of the pond snail *Lymnaea* after predator detection (Il-Han et al., 2010). In *Pleurobranchaea* application of both 5-HT and its precursor, 5-HTP, promote general excitation and decrease feeding thresholds in intact animals (Hirayama et al., 2014).

Previously, we have demonstrated that injections of 5-HT and the precursor 5-HTP into the body of *Helix lucorum* accelerate learning, and also lead to a long-term depolarization of the membrane potential and a decrease of the threshold potential in premotor interneurons for withdrawal when tested days after the injections (Andrianov et al., 2015). A similar acceleration of learning was observed in the leech (Burrell and Sahley, 2005). In contrast, the use of neurotoxic analogs of serotonin 5, 6- or 5,7-DHT, which cause a depletion of 5-HT in synaptic vesicles (Gadotti et al., 1986; Glanzman and Krasne, 1986; Kemenes et al., 1990; Hernádi et al., 1992), blocked the formation of aversive memory (Balaban et al., 1987; Glanzman et al., 1989; Gainutdinov et al., 1999; Andrianov et al., 2015).

A number of studies of the effects of 5-HT application on the electrical characteristics of neurons in a variety of model organisms, including a terrestrial snail, have been conducted. Thus, it was found that the amplitude of EPSPs in premotor interneurons of *Helix* in response to stimulation of the intestinal nerve was increased after bath application of 5-HT in concentrations of 10^−6^–10^−5^ mM, while the input resistance, resting potential and excitability were not changed (Balaban and Zakharov, 1992). It was also shown that incubation of the snail ganglia with 5-HT caused a monotonic decrease in the input resistance of the premotor interneuron LPa3, whereas in sensitized snails the change in the input resistance had a two-phase character: after a relatively short period of decrease in the input resistance, it began to increase (Balaban and Zakharov, 1992). To explain the effect of 5-HT, it was hypothesized that 5-HT causes activation of sodium conductivity and release of calcium from intracellular stores. The effect of 5-HT on neurons was accompanied by depolarization and an increase in input resistance, a decrease in stimulation thresholds, an increase of the amplitude and duration of APs, an increase in the excitability of neurons and a change in the amplitude of ion currents (Klein et al., 1982; Balaban and Zakharov, 1992; Balaban, 1993; Baxter et al., 1999; Barbas et al., 2003; Dumitriu et al., 2006; Jin and Crow, 2011). In *Helix* serotonergic neurons were identified in pedal ganglia that can serve to reinforce associative changes in the efficacy of glutamatergic synaptic inputs to the premotor interneurons that were investigated in the present study (Zakharov et al., 1995; Balaban et al., 2001). These neurons responded to unconditioned stimuli, by releasing serotonin within the neuropile of the parietal ganglia where synapses of premotor interneurons were located, and were necessary for maintenance of aversive context memory (Balaban et al., 2016). Thus, the long-term changes in responsiveness of premotor interneurons could not be due to effects of 5-HT. This fact may be important for the analysis of plasticity changes occurring in the nervous system during learning (Gainutdinov et al., 1998, 2000, 2011; Balaban, 2017). It was also found that prolonged or repeated exposure of the abdominal ganglion to 5-HT leads to rapid hyperexcitability of sensory neurons in *Aplysia* (Liao et al., 1999).

In our study it was found that applications of 5-HT (as well as the precursor of its synthesis 5-HTP) caused a significant decrease in the resting membrane potential (by a mean of 3 mV) of identified premotor interneurons of naïve snails, consistent with an increase in the excitability of these neurons. Most likely, this occurs due to activation of the serotonin type 1 receptors, which are present in the soma of premotor interneurons in snails (Pivovarov and Nistratova, 2003; Abramova et al., 2006; Pivovarov et al., 2014). We also showed that application of 5-HT causes a decrease in the membrane potential (by approximately 4 mV) of premotor interneurons in preparations from the trained snails. Analysis of excitability of these interneurons in trained snails showed that extracellularly applied serotonin or 5-HTP depolarized the investigated neurons, but instead of a decrease of the AP threshold that was observed days after associative training (Figure 3), 30 min of serotonin or 5-HTP bath application elicited an unexpected significant increase of the AP threshold (Figures 4, 5). The functional explanation for these apparently paradoxical results may relate to the homeostatic plasticity phenomena described for the neural circuits (for review see Zenke et al., 2017). Apparently, the homeostatic plasticity observed at the days range is complemented by additional rapid compensatory processes, which rapidly stabilize neuronal activity on short timescales. We can speculate that neurons from the “trained” preparations are already subjected to some homeostatic changes and respond in a different way from neurons from naïve snails to the same stimuli on tens of minutes timescale. At the cellular level it is possible to provide a simplified explanation concerning paradoxical increase in AP thresholds in identified neurons that were subjected to training, resulting in long-term depolarization presumably due to massive serotonin release elicited by electric shock/reinforcement. Further decrease of the AP threshold in “trained” neurons in response to 5-HT/5-HTP applications would lead to constant generation of APs. It is well known that depolarization results in inactivation of a part of voltage-gated sodium channels responsible for AP generation (for review see Catterall, 2012). Changing the balance of different types of ionic channels involved in AP may account for our observation that AP threshold increases in neurons from previously trained animals.

The neurotoxic analog of serotonin 5,7-DHT selectively affects serotonergic elements in the nervous system, causing depletion of 5-HT (Glanzman and Krasne, 1986; Hernádi et al., 1992; Kemenes, 1997). We have previously shown that injection of 5,7-DHT leads to a decrease of the membrane and threshold potentials in the premotor interneurons in snails (Gainutdinov et al., 1999; Andrianov et al., 2015). In this article, we showed that application of 5-HT and 5-HTP to preparations from snails previously injected with 5,7-DHT led to a significant long-term increase of the threshold potential and to decrease of the membrane potential. Thus, 5-HT and 5-HTP affect the premotor interneurons from the 5,7-DHT-injected snails in the same way as in trained snails. These results indicate that training and 5,7-DHT treatment have similar effects. The long-term changes of excitability in these neurons due to an increase of the threshold potential may be due to the influence of extracellular release of serotonin in snails induced by injection of the neurotoxin (Balaban et al., 1987).

## Author Contributions

TKB, IBD and LNM designed and performed behavioral experiments, VVA performed electrophysiological experiments, DIS and AV performed experiments with pharmacology, KLG and PMB designed experiments, estimated significance of effects and wrote the article.

## Conflict of Interest Statement

The authors declare that the research was conducted in the absence of any commercial or financial relationships that could be construed as a potential conflict of interest.

## Abbreviations

5-HT: serotonin
5-HTP: 5-hydroxytryptophan
5,7 DHT: 5,7-dihydroxytryptamine
AP: action potential
CS: conditioned stimulus
SS: saline solution
Vm: membrane resting potential
Vt: threshold potential.
